# Relationship between measures of adiposity, blood pressure and arterial stiffness in adolescents. The MACISTE study

**DOI:** 10.1097/HJH.0000000000003433

**Published:** 2023-05-19

**Authors:** Giacomo Pucci, Maria R. Martina, Elisabetta Bianchini, Marco D’abbondanza, Rosa Curcio, Francesca Battista, Fabio Anastasio, Mariano E. Crapa, Leandro Sanesi, Vincenzo Gemignani, Gaetano Vaudo

**Affiliations:** a Department of Medicine and Surgery, University of Perugia – Unit of Internal Medicine, “Santa Maria” University Hospital, Terni; b Institute of Clinical Physiology, Italian National Research Council, Pisa; c Sports and Exercise Medicine Division, Department of Medicine, University of Padova, Padova; d Cardiology Division, Regina Montis Regalis Hospital, Cuneo; e U.O. Medicina Interna, Asl Taranto, Presidio Ospedaliero Occidentale, Castellaneta, Italy

**Keywords:** adiposity, arterial stiffness, body mass index, carotid stiffness, insulin resistance, neck circumference

## Abstract

Children and adolescents with adiposity excess are at increased risk of future cardiovascular (CV) disease. Fat accumulation promotes the development of elevated blood pressure (BP) and arterial stiffness, two main determinants of CV risk which are strongly inter-related. We aimed at investigating whether the association between overweight and arterial stiffness, taken at different arterial segments, is mediated by increased BP or is BP-independent.

**Methods:**

Three hundred and twenty-two Italian healthy adolescents (mean age 16.9±1.4 years, 12% with overweight) attending the “G. Donatelli” High School in Terni, Italy, underwent measurement of arterial stiffness by arterial tonometry (aortic stiffness) and semiautomatical detection of pressure–volume ratio of the common carotid (carotid stiffness). The mediator effect of BP was tested for each anthropometric or biochemical measure of fat excess related to arterial stiffness.

**Results:**

Both carotid and aortic stiffness showed positive correlations with body mass index, waist, hip, and neck circumferences (NC). Only carotid stiffness, but not aortic stiffness, was associated with serum markers of fat accumulation and metabolic impairment such as insulin, homeostatic model of insulin resistance (HOMA-IR), serum gamma-glutamyl transferase (sGGT) and uric acid. The association with NC was stronger for carotid than for aortic stiffness (Fisher *z*-to-*R* 2.07, *P* = 0.04), and independent from BP.

**Conclusions:**

In healthy adolescents, fat accumulation is associated with arterial stiffness. The degree of this association differs by arterial segments, since carotid stiffness is more strongly associated to adipose tissue excess than aortic stiffness and shows a BP-independent association with NC whereas aortic stiffness does not.

## INTRODUCTION

The rate of children and adolescents with overweight or obesity is increasing worldwide [1]. An excess of adiposity at this early stage of life is acknowledged as a risk factor for the future development of cardiovascular (CV) disease and reduced overall survival [2]. Body fat accumulation promotes the development of an impaired metabolic status characterized by insulin-resistance, increased free fatty acids and ectopic fat deposits [3] that, in turn, paves the way to hypertension, dyslipidaemia and type-II diabetes mellitus [4–6].

An accelerated vascular ageing, expressed by an increase in arterial stiffness, is one of the pathophysiological pathways linking adiposity to future CV risk in children and adolescents [7]. At this age range, the persistence of body fat excess over years, especially if combined with other CV risk factors, has been associated with steeper increases in arterial stiffness over time [8,9]. There are, however, contrasting findings in previous literature regarding the characteristics of the association between fat accumulation and arterial stiffness during adolescence and young adulthood, since this was described as positive by some authors [10–14] but also as negative [9,15–19] by others, leaving unanswered the question whether fat excess is associated with arterial stiffness already at this early stage.

To better address this issue, it should be considered that the physical properties of the arteries are affected not only by the structural characteristics of the vessel wall, such as elastin and collagen content [20], but also by BP values that represent the instantaneous pressure load on the arterial wall [21,22]. Since fat accumulation has been closely linked to increased BP in adolescence [23], to gain further knowledge of the entire process, it is of importance to assess whether and how much, the relationship between fat excess and arterial stiffness is mediated by BP changes or is BP-independent. To this aim, BP must be measured at the same time and site of arterial stiffness measurement [24] and a simultaneous noninvasive central BP estimation is required to evaluate the impact of BP components, such as systolic BP or PP, on stiffness of central arteries such as the aorta and the carotid arteries.

It should also be considered that measures of arterial stiffness from different arterial segments (e.g. aortic stiffness and carotid stiffness) could variably respond to the effects of metabolic and CV risk factors [25], and that some measures of local adiposity, such as waist or neck circumference (NC), could be more closely associated to increased arterial stiffness than traditional indexes of global obesity (e.g. BMI) [26]. Furthermore, based on previous findings, a comprehensive evaluation of clinical manifestations of fat-related metabolic impairment in their association with arterial stiffness among adolescents should also include the analysis of biochemical markers of adipose excess and metabolic derangement, such as insulin serum levels, homeostatic model of insulin resistance (HOMA-IR), serum gamma glutamyltransferase (sGGT) and uric acid [27–29].

To take due account all these aspects, with the aim of gaining better knowledge on the association between fat accumulation and various measures of arterial stiffness, and to properly assess the mediating role of BP in such association, we analysed data from a cohort of Italian healthy adolescents undergoing an extensive evaluation of cardiovascular and metabolic status enrolled in the MACISTE study [30]. In the present study we analysed the relationship between arterial and carotid stiffness with measures of global and local adipose tissue excess and laboratory markers of fat-related metabolic impairment, using noninvasive estimates of central BP values to assess the mediating role of central BP.

## METHODS

### Participants

Details of the MACISTE study were reported elsewhere [30]. Briefly, a cohort of healthy Italian scholars of the “G. Donatelli” High School in Terni, Italy, aged 13–19 years old, underwent an extensive anthropometric, clinic, laboratory and instrumental evaluation. The study took place within the high school premises and was conducted between March and June 2015.

Participants and their parents (parents’ signature was optional for individuals aged ≥18 years old) were notified about study procedures and were asked to give informed consent. All measurements and procedures were carried out at the high school, in order to provide the best approach for motivating individuals to participate to the project. The present study was approved by the Local Ethic Committee and conducted in accordance with the Declaration of Helsinki.

### Measurements

All measurements were performed on each participant within the same day. All participants were evaluated during the early morning and after at least 13 h of overnight fasting. Smoking or caffeine consumption was not allowed during the day before.

Blood samples were drawn after 13-h overnight fasting conditions and delivered to the centralized laboratory of the Terni University Hospital for the analysis. Low-density lipoprotein (LDL) cholesterol was calculated by the Friedewald formula. Fasting serum insulin, the HOMA-IR calculated from the model described by Matthews *et al.*[31], serum uric acid (SUA) and sGGT were analysed as laboratory markers related to metabolic status.

Body weight was measured to the nearest 0.5 kg, with participants wearing light clothing and no shoes. Height was measured to the nearest 0.5 cm. BMI was calculated as follows: weight (in kg)/height (in m^2^) and expressed as percentile values based on age and sex, according to the Centers for Disease Control and Prevention (CDC) Growth Charts. Overweight was defined as BMI ≥85° percentile or as BMI >25 kg/m^2^[32]. Waist circumference (WC) was measured on a horizontal plane with an inextensible flexible measuring tape to the nearest 0.1 cm at the mid-point between the lowest floating rib and the upper border of iliac crest at the end of a normal expiration. Hip circumference (HC) was taken at the maximum protuberance of the buttocks. NC was measured below the cricoid cartilage, at the level of the mid-cervical spine. Waist-to-hip ratio (WHR) and waist-to-height ratio were also calculated as measures of body fat distribution.

### Blood pressure and arterial stiffness

BP was measured by a validated oscillometric device for children and adolescents (Omron 705 IT) [33], as previously described. All participants rested in recumbent supine position for at least 10 min. Subsequently, carotid-to-femoral pulse wave velocity (cf-PWV), a surrogate measure of aortic stiffness, or carotid stiffness was measured in a random order in each participant. The first measurement was preceded by three BP measurements, whose values were averaged and used for subsequent analysis. Changes in body posture were avoided during all the procedure. Cf-PWV was measured with the SphygmoCor system (SphygmoCor Vx; AtCor Medical, Sydney, Australia) through high-fidelity applanation tonometer [34]. The path length was calculated as 0.8 × the direct distance between the two arterial sites measured directly with a calliper. Radial and brachial pulse waveforms were also detected by applanation tonometry. Brachial pulse waveform was then calibrated to brachial systolic and diastolic BPs (SBP/DBP) to obtain a measure of brachial mean arterial pressure (MAP) by the area-under-the-curve (AUC) method [35]. Pulse pressure (PP) was calculated as SBP − DBP. Central (aortic) pressure waveform was derived from the radial waveform using the in-built SphygmoCor generalized transfer function, and calibrated using brachial MAP and DBP to obtain aortic SBP and PP, under the assumption that these two measures are rather constant along the arterial tree. Carotid pressure waveform was calibrated to brachial MAP and DBP to obtain carotid SBP and PP.

Carotid stiffness was assessed by means of the Medical Device Software Carotid Studio (Cardiovascular Suite, Quipu srl, Pisa, Italy) which allows the estimation of vascular parameters by ultrasound imaging, based on a validated contour tracking algorithm [36,37]. The system processed B-mode longitudinal acquisitions of common carotid artery (video frame rate = 25 frames/s) where the arterial interfaces were automatically detected flanked by the estimation of instantaneous diameter as the distance between far and near media–adventitia interfaces. Systolic and diastolic diameters were detected for each cardiac cycle and carotid distension, the stroke change in diameter, was calculated as the mean value of their difference on common carotid artery images sequence (video duration = 10 s). Stroke change in lumen area (Δ*A*) and lumen area (*A*), which is the diastolic lumen area evaluated from the diameter values assuming that the cross-section of the artery is circular, were also calculated. The instantaneous diameters flanked by carotid SBP/DBP were used to calculate carotid stiffness (CS). Specifically, the cross-sectional distensibility coefficient was calculated as DC = Δ*A*/(*A* × PP), where PP is carotid PP. Bramwell–Hill equation, derived by the Moens–Korteweg equation, converted DC into a carotid stiffness parameter according to the relationship CS = (DC × *ρ*)(−1/2), where *ρ* represents the blood density. CS was expressed with the same measurement units of pulse wave velocity (m/s) thus enabling a direct comparison between the two techniques [38].

### Statistical analysis

The assumption of satisfactory normal distribution was tested for all the examined variables by the Kolmogorov–Smirnov *Z* test. Continuous variables were expressed as mean ± standard deviation (SD) if normally distributed, or as median and interquartile range (IQR) if nonnormally distributed. Categorical variables were expressed by their relative (%) frequencies. If nonnormally distributed, variables were log-transformed before entering into linear models. Pearson's *R* and Spearman's rho correlation coefficients were used to describe the correlation between parametric and nonparametric variables. The Fisher *r*-to-*z* transformation was applied to determine the significance of the difference between two correlation coefficients. Given that a sex-specific susceptibility of weight induced arterial stiffening and BP rise was described in previous literature [39], we tested the significance of the interaction term ‘sex’ on the association between fat-related variables and measures of arterial stiffness.

Multiple adjustments were made through stepwise linear multivariate models where carotid stiffness and cf-PWV were introduced as dependent variables in separate models. In each model, a measure of body fatness was introduced (one at a time) along with age, sex, heart rate, total cholesterol, Tanner state and levels of physical activity. Further adjustment was also made by introducing also brachial MAP or (aortic or carotid) PP to the fully adjusted models. Before performing multivariate analyses, all variables were tested for collinearity through appropriate statistics.

To address the mediating effect of BP in the relationship between fat accumulation and arterial stiffness, we first run the Sobel's test [40], which was further refined using the bootstrapping procedure provided in the SPSS PROCESS 3.4 version (SPSS version 22, IBM Corp., Armonk, New York, USA) in order to account for potential bias related to small sample size. In this analysis, the direct, indirect, and total effects were calculated using the product of coefficients strategy and tested using the bootstrapping method (*N* = 5000). If the 5000 bootstrap 95% bias-corrected confidence intervals (CIs) did not cross 0, the indirect effect was taken as significant. The covariates of age, sex, height, heart rate, physical activity and Tanner status were introduced as independent variables in the mediation models. *P* < 0.05 (two-tailed) was considered statistically significant. Assuming from previous literature [11] that the total effect of fat accumulation on arterial stiffness parameters is the sum of direct and indirect effects, both contributing to the same extent to the outcome variable, we hypothesized that a sample size of 346 patients would provide the 80% power with a significance criterion of *α* = 0.05.

## RESULTS

From an initial population of 539 participants, 44 individuals were excluded due to low quality of arterial waveform signal by applanation tonometry, 64 individuals were also excluded due to low quality carotid ultrasound images, and 109 individuals refused to undergo blood test.

### Measures of adiposity and blood pressure

Three hundred and twenty-two participants (mean age 17 ± 1.4 years, 56% boys) were evaluated, 40 (12%) were found with overweight. The clinical, anthropometric and laboratory characteristics of the study population according to overweight status are summarized in Table 1. There were no differences in sex distribution between participants with overweight and normal weight (*P* = 0.89). As expected, all anthropometric and biochemical measures related to fat excess were higher in participants with overweight than normal weight (all *P* < 0.05) with the exception of HDL-cholesterol, which was lower in participants with overweight than in normal weight (*P* = 0.04), and glucose and triglycerides serum levels, which were not significant by groups.

**TABLE 1:** Characteristic of study population by overweight status

	Total	Overweight	Normal weight	*P*
*N* (%)	322	40 (12)	282 (88)	–
Male sex, *n* (%)	181 (56)	23 (58)	158 (56)	0.89
Age, years	16.9 (1.4)	16.9 (1.4)	16.9 (1.4)	0.91
Height, cm	170 (9)	169 (9)	170 (9)	0.39
Weight, kg	62 (11)	76 (11)	60 (9)	<0.001
BMI, kg/m^2^	21.4 (3)	26.8 (2)	20.7 (1)	<0.001
Waist circumference, cm	78 (9)	90 (9)	76 (8)	<0.001
Hip circumference, cm	95 (7)	105 (6)	93 (6)	<0.001
Neck circumference, cm	33 (3)	35 (3)	32 (3)	<0.001
Waist-to-height ratio	0.46 (0.05)	0.53 (0.05)	0.45 (0.04)	<0.001
Waist-to-hip ratio	0.82 (0.05)	0.85 (0.06)	0.81 (0.06)	<0.01
Brachial SBP, mmHg	124 (11)	130 (12)	123 (11)	<0.001
Brachial DBP, mmHg	67 (7)	70 (8)	66 (7)	<0.01
Brachial PP, mmHg	57 (10)	60 (11)	57 (10)	0.10
Mean arterial pressure, mmHg	86 (8)	91 (9)	86 (8)	<0.001
Aortic SBP, mmHg	105 (9)	112 (9)	104 (8)	<0.001
Aortic DBP, mmHg	69 (8)	72 (8)	68 (8)	<0.01
Aortic PP, mmHg	36 (7)	40 (8)	36 (7)	<0.01
PP amplification,	1.57 (0.13)	1.51 (0.13)	1.58 (0.13)	<0.01
Carotid-femoral PWV, m/s	4.9 (0.8)	5.0 (0.7)	4.9 (0.8)	0.77
Carotid stiffness, m/s	4.3 (0.6)	4.5 (0.6)	4.2 (0.6)	0.01
Total cholesterol, mg/dl	160 (30)	171 (35)	159 (29)	0.04
HDL-cholesterol, mg/dl	55 (11)	52 (9)	56 (11)	0.04
LDL-cholesterol, mg/dL	92 (32)	105 (37)	90 (31)	0.02
Serum triglycerides, mg/dl	59 (49–77)	61 (50–77)	59 (49–77)	0.67
Serum glucose, mg/dl	83 (8)	84 (7)	83 (8)	0.71
Serum insulin, mmol/l	11.6 (8.7–14.5)	15.1 (12.4–19.8)	11.1 (8.6–14.1)	<0.001
HOMA-IR	2.4 (1.8–3.0)	3.0 (2.4–4.2)	2.3 (1.7–2.9)	<0.001
Serum uric acid, mg/dl	5.6 (1.3)	6.1 (1.6)	5.5 (1.2)	0.02
sGGT, U/l	16 (13–20)	18 (14–27)	16 (13–19)	0.03

Results are presented as mean (SD) or as median (25°–75° IQR) where applicable. BMI, body mass index; DBP, diastolic blood pressure; HDL, high-density lipoprotein; HOMA-IR, homeostatic model assessment-insulin resistance; LDL, low-density lipoprotein; PP, pulse pressure; PWV, pulse wave velocity; sGGT, serum gamma glutamyltransferase; SBP, systolic blood pressure.

Measures of central (aortic) and peripheral BP were higher in participants with overweight as compared to normal weight (all *P* < 0.01), with the exception of peripheral and central DBP, which were significantly lower in overweight vs. normal weight, and brachial PP, which was comparable between the two groups. The aortic-to-brachial PP amplification was reduced in participants with overweight than in lean participants (1.51 ± 0.13 vs. 1.58 ± 0.13, *P* < 0.01). When the association between the examined variables and overweight status was analysed according to sex (Table 1, Supplemental Digital Content), the main results did not substantially change. Specifically, sex was not an effect modifier of the relationship between all the examined variables and overweight status (all *P* for sex-interaction >0.05).

### Measures of adiposity and arterial stiffness

**FIGURE 1:**
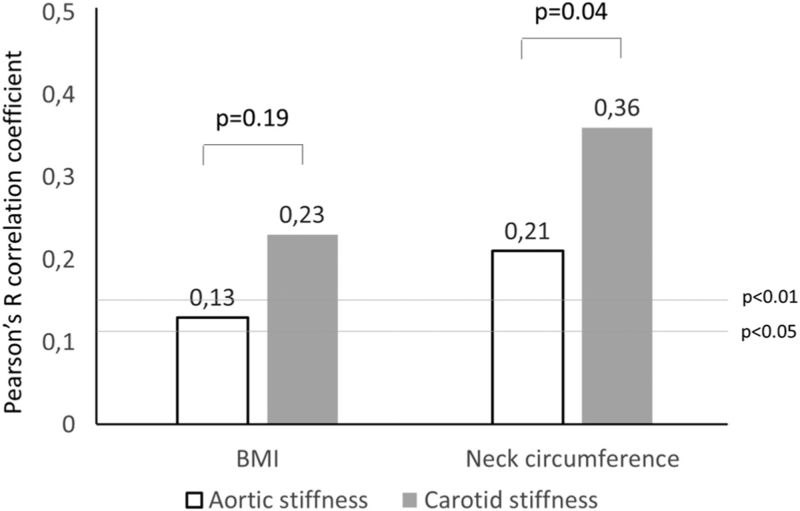
Pearson's *R* correlation coefficients between BMI and neck circumference. *P*-values above the columns represent the significance of the Fisher *r*-to-*z* transformation. *P*-values on the horizontal lines represent the two levels of significance of *R*.

Both carotid and aortic stiffness showed a positive correlation with BMI and with measures of local adiposity such as WC and HC, WHR and NC (Table 2). The correlation between NC and aortic stiffness (cf-PWV) was significantly weaker than the correlation with carotid stiffness (Fisher *z*-to-*R* 2.07, *P* = 0.04, Fig. 1). Only carotid stiffness, but not aortic stiffness, was positively associated with markers of impaired glyco-metabolic status such as serum insulin, HOMA-IR, sGGT and SUA. Sex did not significantly affect the strength of the association between any parameter of adiposity or impaired metabolic profile with measures of arterial stiffness (all *P* for sex-interaction >0.05).

**TABLE 2:** Correlations between cf-PWV, carotid stiffness and measures of fat accumulation

	Cf-PWV	Carotid stiffness	*P* (*r*-to-*z*)
BMI	0.13^∗^	0.23^∗∗^	0.19
Waist circumference	0.15^∗∗^	0.22^∗∗^	0.35
Hip circumference	0.09	0.16^∗∗^	0.37
Neck circumference	0.21^∗∗^	0.36^∗∗^	0.04
Waist-to-height ratio	0.09	0.15^∗∗^	0.37
Waist-to-hip ratio	0.13^∗^	0.17^∗∗^	0.60
Total cholesterol	0.12	0.12	0.98
HDL-cholesterol	−0.01	−0.06	0.52
LDL-cholesterol	0.11	0.12	0.90
Triglycerides	0.03	0.08	0.53
Serum glucose	−0.07	0.06	0.08
Serum insulin	−0.06	0.17^∗∗^	<0.01
HOMA-IR	−0.07	0.18^∗∗^	<0.01
Serum uric acid	0.07	0.15^∗^	0.31
sGGT	0.05	0.22^∗∗^	0.03

Numbers represented Pearson's *R* (for parametric variables) or Spearman's rho (for nonparametric variables) correlation coefficients between each variable, cf-PWV (carotid-femoral pulse wave velocity) and carotid stiffness. ^∗^*P* < 0.05, ^∗∗^*P* < 0.01. *P* (*r*-to-*z*): *P*-values of the Fisher *r*-to-*z* transformation to determine the significance of the difference between the two correlation coefficients. BMI, body mass index; cf-PWV, carotid-femoral pulse wave velocity; HDL, high-density lipoprotein; HOMA-IR, homeostatic model assessment-insulin resistance; LDL, low density lipoprotein; sGGT, serum gamma glutamiltransferase.

### Multivariate analyses

The correlation between measures of adiposity, metabolic status, BP and arterial stiffness were tested in separate multivariate models. In each mode, a measure of arterial stiffness (aortic, carotid) was introduced as the dependent variable, and each measure of adiposity or metabolic status (one per each model), together with other fixed independent variables following three steps. In the first step (Model 1, Table 3) age (in months), sex, height, heart rate, Tanner stages and level of physical activity were included. Then, MAP was added to the model in the second step (Model 2); finally, MAP was replaced with PP in the third step (Model 3).

**TABLE 3:** Multivariate analysis investigating the main determinants of carotid stiffness (upper part) and aortic stiffness (lower part)

	Model 1		Model 2		Model 3	
Carotid stiffness						
	*β*	*P*	*β*	*P*	*β*	*P*
BMI	0.17	<0.01	0.07	0.13	0.03	0.60
Waist circumference	0.13	<0.01	0.04	0.36	0.02	0.63
Hip circumference	0.17	<0.01	0.08	0.09	0.02	0.59
Waist/hip ratio	0.04	0.45	0.02	0.75	0.01	0.83
Neck circumference	0.35	<0.01	0.24	<0.01	0.15	0.04
Serum insulin	0.10	0.06	0.02	0.71	0.06	0.20
HOMA-IR	0.13	0.03	0.06	0.24	0.08	0.13
Serum uric acid	0.05	0.40	0.02	0.78	0.02	0.69
sGGT	0.05	0.39	0.04	0.41	0.03	0.51
Aortic stiffness						
BMI	0.06	0.14	0.03	0.51	0.03	0.62
Waist circumference	0.10	0.03	0.07	0.13	0.01	0.87
Hip circumference	0.07	0.09	0.04	0.38	0.01	0.86
Waist/hip ratio	0.07	0.11	0.06	0.18	0.08	0.10
Neck circumference	0.17	<0.01	0.16	0.02	0.12	0.07
Serum insulin	0.01	0.96	0.01	0.83	0.01	0.75
HOMA-IR	0.06	0.25	0.02	0.79	0.01	0.97
Serum uric acid	0.02	0.70	0.04	0.42	0.03	0.63
sGGT	0.07	0.21	0.07	0.17	0.05	0.35

Each line represented a single multivariate model in which a measure of adiposity or metabolic status was introduced as independent variable together with other fixed variables: age, sex, height, heart rate, Tanner stages, level of physical activity in Model 1; variables in Model 1 plus MAP in Model 2; variables in Model 1 plus (aortic or carotid) PP in Model 3. BMI, body mass index; HOMA-IR, homeostatic model assessment-insulin resistance; sGGT, serum gamma glutamiltransferase.

Many of the observed univariate associations of carotid and aortic stiffness with measures of adiposity lost its significance when MAP was introduced to the model (Model 2). Only NC remained associated with carotid (*β* = 0.24, *P* < 0.01) and aortic stiffness (*β* = 0.16, *P* = 0.02). When MAP was replaced with carotid PP (Model 3), NC was the only measure to remain associated with carotid stiffness (*β* = 0.15, *P* = 0.04), whereas its association with aortic stiffness lost significance (*β* = 0.12, *P* = 0.07).

### Mediation analysis

**FIGURE 2:**
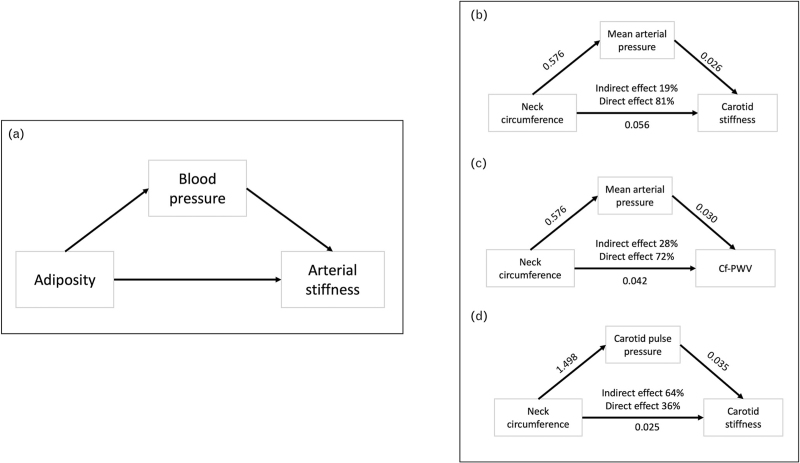
Mediation analysis. (a) General conceptual model. (b) Mediator effect of mean arterial pressure on the relationship between neck circumference and carotid stiffness. (c) Mediator effect of mean arterial pressure on the relationship between neck circumference and carotid-femoral pulse wave velocity (cf-PWV). (d) Mediator effect of pulse pressure on the relationship between neck circumference and carotid stiffness. Numerical values represent unstandardized regression coefficients.

In a mediation analysis model, built on the general conceptual model that adiposity could negatively be associated with arterial stiffness through direct effects and through increased BP (Fig. 2), MAP was found to be a significant mediator of the association between NC and cf-PWV (Sobel test 2.70, *P* < 0.01, direct effect 81%, indirect effect 19%, Fig. 2), and between NC and carotid stiffness (Sobel test 2.27, *P* < 0.01, direct effect 72%, indirect effect 28%). NC remained significantly associated with carotid stiffness even after accounting for the mediating effect of carotid PP (Sobel test 1.98, *P* = 0.04, direct effect 36%, indirect effect 64%), although the strength of this association was very poor. The bootstrapping analysis overall confirmed these results (Table 3, Supplemental Digital Content).

## DISCUSSION

In our study we showed that, in a cohort of healthy adolescents, indexes of body fat accumulation such as BMI, WC, HC, waist/hip ratio and NC were positively associated with measures of aortic and carotid stiffness. Carotid stiffness showed overall more robust associations with body fat accumulation than aortic stiffness, as it was demonstrated for NC, and was also significantly associated with laboratory biomarkers of impaired metabolic status, such as serum insulin, HOMA-IR, SUA and sGGT, whereas aortic stiffness did not.

Our study investigated the mediating role of BP in the association between fat accumulation and arterial stiffness in adolescents. In line with previous literature, showing that fat excess was closely linked to increased BP in adolescence [23], we demonstrated that BP is a key mediator of the association between neck circumference, a measure of fat accumulation largely used among adolescents, and aortic and carotid stiffness. As a novel interesting finding, we observed that the direct association between NC and carotid stiffness, although very poor, remained significant after considering the mediating role of BP either taken as MAP, a measure of distending pressure, or as carotid PP. Given that arterial stiffness is the result of both pressure load-dependent and load-independent components (structural stiffness) [20], and that these latter might be negatively affected by fat excess, our results suggest that carotid stiffness is a marker of fat-mediated organ damage, independently from BP values, in adolescents.

In comparison to previous findings (Table 2, Supplemental Digital Content), the novelty of our approach was that each measure of arterial stiffness was evaluated not only according to MAP values, which are known to be constant along the arterial tree, but also according to measures of local pulsatility (e.g. aortic and carotid PP) to eliminate the possible residual confounding effect related to overweight status. It has been described, indeed, that overweight in adolescence is an amplifier of the PP increase that follows body growth [23]. Interestingly, the association between NC and carotid stiffness remained significant after accounting for the mediating role of increased carotid PP, whereas the association between NC and aortic stiffness was no longer significant after adjustment for aortic PP.

Our mediation analysis was based on the general conceptual model that adiposity in adolescence is a key antecedent of both elevated BP and increased arterial stiffness. Although limited by the cross-sectional nature, this model was supported by results from previous animal experiments [41], human observational prospective large-scale studies [9] and lifestyle interventions aiming at reducing fat deposits, such as weight reduction or physical exercise [42,43] that demonstrated that fat excess is associated with increased BP and arterial stiffness and that BP and arterial stiffness could be reduced by targeted lifestyle interventions.

Our results, if viewed within the context of previous literature, generally confirm that the aortic and carotid arterial pathways differently respond to the detrimental effect of excess adiposity. Paini *et al.*[44] first demonstrated that the strength of association between measures of carotid and aortic stiffness decreased at increasing number of CV risk factors, thus first showing a differential sensitivity of various arterial pathways to factors affecting the elastic behaviour. Mikola *et al.*[45] evaluated trajectories of aortic and carotid distensibility from childhood to early adulthood by ultrasound assessment. Despite inherent methodological differences, they were able to demonstrate that carotid distensibility was the only measure of arterial function to be related to BMI and HOMA-IR. Zachariah *et al.*[46] found that, in a sample of normal weight and overweight adolescents, significant positive associations between BMI and arterial stiffness that became weaker at increasing arterial size, as it is the case of aortic, compared to carotid, dimensions. Finally, Vriz *et al.*[47] showed that carotid, but not aortic, stiffness was significantly associated with measures of left ventricular (LV) structure and function.

Other results deserve to be commented in our study. We found that participants with overweight showed a reduced PP amplification, thus suggesting that adiposity is associated with increased PP more strongly at the central level than in periphery. This feature also confirms that adiposity has a negative impact on arterial function through its effects on central large arteries [48]. In our population of healthy Italian adolescents, a relatively high proportion of adolescents was found with overweight (12%). These participants frequently showed an impaired lipid profile, increased insulin-resistance, elevated SUA levels and laboratory signs of metabolic derangement, strongly supporting the evidence that adiposity excess could negatively impact on the glyco-metabolic status even at this early stage, as it was observed in previous literature [49]. Contrary to previous findings observed in adult population [39], our results did not show the role of sex as a significant effect modifier of the association between obesity, increased BP and arterial stiffness in adolescents.

Overall, these results could inform targeted therapeutic interventions to young individuals, in the setting of primary prevention with the aim of reducing risk of future CV disease. Our study also confirms previous evidences in favour of the evaluation of NC as a proxy measure of metabolic impairment and unhealthy CV health in adolescence, supporting its use in clinical practice due to its simplicity of measurement [50], and also raising interesting hypotheses on the paracrine role of compartmental fat of the neck as a direct modifier of the carotid artery wall mechanics.

Strengths of our study were the use of validated and up-to-date technologies for the assessment of aortic and carotid stiffness and the availability anthropometric and laboratory parameters of body fat accumulation and metabolic status, combined with accurate noninvasive estimates of BP at the level of arterial stiffness measurement. It should be acknowledged, however, that in our study the use of different technologies for the assessment of aortic and carotid stiffness might have impacted on results. Carotid stiffness was measured locally at a single arterial point through a nonpropagative model based on the volume/pressure relationship of a cross-sectional arterial segment. Aortic stiffness was measured by the propagation velocity of the pulse wave along two arterial points, classically along the carotid-femoral segment. Previous studies reported that different methodologies for arterial stiffness assessment could provide different results because of different BP and HR dependencies [25]. As previously stated, the cross-sectional nature of our study does not allow to determine the causal relationship between variables. In this regard, the hypothesis that elevated BP is already the phenotypic expression of an increased arterial stiffness, as it was recently proposed [46], cannot be a priori ruled out. Further prospective observations are needed to define the direction of such associations in adolescents.

We acknowledge that metrics adopted for defining overweight derived from a population percentile distribution approach rather than the association with disease outcomes. Despite we attempted to select variables reflecting body fat distribution to those with documented relationship with insulin resistance and CV risk, we could not exclude that other biological or behavioural components might display stronger association with arterial stiffness than those documented in our study. Anatomical and structural neck differences could have also impacted on results; in order to minimize this effect and to reduce variability, a validated medical device software (Quipu srl) based on a robust contour-tracking algorithm was adopted flanked by properly set and standardized ultrasound measurement protocols. Finally, the small sample size, also determined by the lower than the expected number of participants, requires that results of our study should be confirmed in larger datasets.

In conclusion, in line with previous literature, our results support the view that adiposity is a determinant of early vascular ageing in adolescence, and that increased BP is a significant mediator of such association. Our results could therefore raise the hypothesis that therapeutic interventions aimed at reducing the burden of increased adiposity in adolescents could positively impact on CV health through preservation of normal arterial function and reduction of BP values.

## ACKNOWLEDGEMENTS

The authors express their deepest gratitude to Professor Giuseppe Schillaci for his inestimable contribution to the conception, design and development of the MACISTE Study.

Sources of funding: The MACISTE study was funded by “Regione Umbria − research project for programming health, prevention of chronicity and model organization in the context of dysmetabolical diseases”, year 2013

### Conflicts of interest

E.B. and V.G. are co-founders of QUIPU s.r.l., Pisa, Italy, a spin-off company of the Italian National Research Council and the University of Pisa developing software medical devices.

## Supplementary Material

Supplemental Digital Content
