# Supplementary material for: Relationship between measures of adiposity, blood pressure and arterial stiffness in adolescents. The MACISTE study
Source: J Hypertens. 2023 May 19;41(7):1100–7. doi: 10.1097/HJH.0000000000003433 (PMC10241423; doi:10.1097/HJH.0000000000003433)
Supplement: Supplemental Digital Content [file jhype-41-1100-s001.docx]

**Relationship between measures of adiposity, blood pressure and arterial stiffness in adolescents. The MACISTE study**

*Running head: adiposity and arterial stiffness in young*

Giacomo PUCCI ^1^

Maria R. MARTINA ^2^

Elisabetta BIANCHINI ^2^

Marco D’ABBONDANZA ^1^

Rosa CURCIO ^1^

Francesca BATTISTA ^3^

Fabio ANASTASIO ^4^

Mariano E. CRAPA ^5^

Leandro SANESI ^1^

Vincenzo GEMIGNANI ^2^

Gaetano VAUDO ^1^

^1^ Department of Medicine and Surgery, University of Perugia - Unit of Internal Medicine, "Santa Maria" University Hospital, Terni, Italy.

^2^ Institute of Clinical Physiology, Italian National Research Council, Pisa, Italy.

^3^ Sports and Exercise Medicine Division, Department of Medicine, University of Padova, Padova, Italy.

^4^ Cardiology Division, Regina Montis Regalis Hospital, Cuneo, Italy.

^5^ U.O. Medicina Interna, Asl Taranto, Presidio Ospedaliero Occidentale, Castellaneta, Italy.

**Supplementary Tables**

**Supplementary Table 1.** Characteristic of study population by sex and overweight status

|  | Boys | | | Girls | | | P (boys vs girls) | P (sex interaction) |
| --- | --- | --- | --- | --- | --- | --- | --- | --- |
|  | Overweight | Normal weight | P | Overweight | Normal weight | P |  |  |
| N (%) | 23 (7) | 158 (49) | - | 17 (5) | 124 (39) | - |  |  |
| Age, years | 16.9 (1.3) | 16.8 (1.3) | 0.67 | 17.0 (1.5) | 16.9 (1.4) | 0.76 | 0.84 | 0.60 |
|  | | | | | | | | |
| ***Measures of body size and adiposity*** | | | | | | | | |
| Height, cm | 174 (7) | 174 (7) | 0.78 | 161 (6) | 164 (6) | 0.07 | <0.001 | 0.19 |
| Weight, kg | 82 (10) | 64 (9) | <0.001 | 69 (7) | 55 (7) | <0.001 | <0.001 | 0.09 |
| BMI, kg/m^2^ | 27.0 (2) | 20.9 (2) | <0.001 | 26.6 (2) | 20.3 (2) | <0.001 | 0.07 | 0.96 |
| Waist circumference, cm | 93 (9) | 78 (7) | <0.001 | 86 (7) | 74 (8) | <0.001 | <0.001 | 0.18 |
| Hip circumference, cm | 105 (7) | 93 (6) | <0.001 | 106 (5) | 94 (6) | <0.001 | 0.72 | 0.42 |
| Neck circumference, cm | 37 (2) | 34 (2) | <0.001 | 32 (2) | 30 (2) | <0.001 | <0.001 | 0.82 |
| Waist-to-height ratio | 0.53 (0.05) | 0.45 (0.04) | <0.001 | 0.54 (0.04) | 0.45 (0.04) | <0.001 | 0.53 | 0.18 |
| Waist-to-hip ratio | 0.88 (0.05) | 0.84 (0.05) | <0.001 | 0.82 (0.07) | 0.79 (0.07) | 0.18 | <0.001 | 0.07 |
|  | | | | | | | | |
| Blood pressure measurements | | | | | | | | |
| Brachial SBP, mmHg | 136 (8) | 127 (10) | <0.001 | 122 (11) | 117 (10) | 0.07 | <0.001 | 0.30 |
| Brachial DBP, mmHg | 69 (7) | 65 (7) | 0.02 | 72 (9) | 68 (7) | 0.03 | <0.01 | 0.97 |
| Brachial PP, mmHg | 66 (8) | 62 (9) | 0.02 | 50 (7) | 50 (8) | 0.81 | <0.001 | 0.25 |
| Mean arterial pressure, mmHg | 92 (8) | 86 (8) | 0.001 | 90 (10) | 85 (8) | 0.03 | 0.65 | 0.52 |
| Aortic SBP, mmHg | 115 (7) | 107 (8) | <0.001 | 107 (11) | 101 (8) | 0.01 | <0.001 | 0.16 |
| Aortic DBP, mmHg | 71 (7) | 67 (8) | 0.03 | 74 (9) | 70 (7) | 0.04 | 0.01 | 0.94 |
| Aortic PP, mmHg | 44 (6) | 39 (6) | <0.001 | 33 (5) | 32 (5) | 0.20 | <0.001 | 0.26 |
| PP amplification, | 1.51 (0.12) | 1.58 (0.13) | 0.02 | 1.51 (0.14) | 1.58 (0.13) | 0.04 | 0.85 | 0.89 |
|  | | | | | | | | |
| ***Arterial stiffness parameters*** | | | | | | | | |
| Carotid-femoral PWV, m/s | 5.1 (0.8) | 5.0 (0.9) | 0.80 | 4.8 (0.5) | 4.7 (0.7) | 0.91 | <0.001 | 0.95 |
| Carotid stiffness, m/s | 4.6 (0.5) | 4.4 (0.6) | 0.04 | 4.3 (0.6) | 4.1 (0.6) | 0.05 | <0.001 | 0.78 |
|  | | | | | | | | |
| ***Laboratory variables*** | | | | | | | | |
| Total cholesterol, mg/dL | 165 (24) | 151 (28) | 0.04 | 179 (46) | 171 (27) | 0.41 | <0.001 | 0.48 |
| HDL-cholesterol, mg/dL | 49 (8) | 55 (11) | 0.02 | 56 (9) | 57 (11) | 0.77 | 0.06 | 0.16 |
| LDL-cholesterol, mg/dL | 102 (24) | 83 (30) | 0.01 | 109 (51) | 101 (30) | 0.41 | <0.001 | 0.27 |
| Serum triglycerides, mg/dL | 64 (50 – 83) | 60 (47 – 78) | 0.48 | 57 (50 – 73) | 58 (49 – 77) | 0.90 | 0.68 | 0.21 |
| Serum glucose, mg/dL | 86 (7) | 83 (7) | 0.06 | 80 (6) | 83 (8) | 0.12 | 0.12 | 0.02 |
| Serum insulin, mmol/L | 15.7 (12.8 – 22.3) | 10.8 (8.3 – 13.1) | <0.001 | 14.7 (12.0 – 19.5) | 11.9 (8.6 – 15.0) | 0.02 | 0.10 | 0.28 |
| HOMA-IR | 3.2 (2.4 – 4.8) | 2.2 (1.7 – 2.8) | <0.01 | 2.9 (2.3 – 3.5) | 2.4 (1.8 – 3.2) | 0.05 | 0.29 | 0.43 |
| Serum Uric acid, mg/dL | 6.9 (1.2) | 6.0 (1.1) | <0.01 | 5.0 (1.6) | 4.8 (1.0) | 0.46 | <0.001 | 0.26 |
| sGGT, U/L | 20 (17 – 29) | 17 (15 – 21) | <0.01 | 14 (12 – 15) | 13 (11 – 16) | 0.29 | <0.001 | 0.08 |

**Supplementary Table 2.** Summary of results of previous results describing the relationship between measures of fat excess and arterial stiffness parameters.

| First author | Year | Population | Measure of fat excess | Arterial stiffness parameter | Direction of the association with arterial stiffness | Adjusted for |
| --- | --- | --- | --- | --- | --- | --- |
| Cruickshank JK [10] | 2016 | 666 young adults (21-23 years) | Waist-height ratio | PWV | Positive | Brachial BP |
| Fernberg U [11] | 2019 | 220 healthy individuals (18.-25 years) | BMI, body fat %, waist circumference | Carotid stiffness | Positive | Brachial MAP |
| Juonala M [12] | 2005 | 2187 children and adolescents (3-18 years) | Skinfold thickness | Carotid stiffness | Positive | Brachial SBP |
| Tounian L [13] | 2001 | 48 severely obese children (3-15 years), 27 controls | Total body fat, BMI z-score, android/gynoid FMR | Carotid stiffness | Positive | Unadjusted |
| Zebekakis PE [14] | 2005 | 1306 individuals (10-86 years) | BMI at 20 years | Carotid stiffness | Positive | Brachial MAP |
| Lurbe E [15] | 2012 | 501 individuals (8-18 years) | BMI z-score | Carotid-femoral PWV | Negative | Central SBP |
| Corden B [16] | 2013 | 221 individuals (18-72 years) | Percentage body fat (20 years) | Carotid-femoral PWV | Negative | Brachial MAP |
| Dangardt F [17] | 2008 | 31 obese children, 18 lean controls (14±2 years) | BMI z-score | Carotid-radial PWV | Negative | Unadjusted |
| Charakida M [18] | 2012 | 169 obese children (10 years) | BMI | Carotid-radial PWV | Negative | Brachial SBP |
| Donald AE [19] | 2010 | 6814 children (10 - 12 years) | BMI | Carotid-radial PWV | Negative | Brachial SBP and DBP |
| Donald AE [19] | 2010 | 7209 children (10 – 12 years) | BMI | Carotid stiffness | Negative | Brachial SBP and DBP |
| Dangardt F [9] | 2019 | 1910 Individuals (17 years) | Total fat mass, Kg | Carotid-radial PWV | Positive | Brachial SBP |
| Pucci G | 2023 | 322 healthy adolescents | Neck circumference | Carotid-femoral PWV | Not singificant | Central PP |
| Pucci G | 2023 | 322 healthy adolescents | Neck circumference | Carotid stiffness | Positive | Central PP |

The last two lines refer to results of the present study. References are reported in square brackets. BMI: body mass index. FMR: fat mass ratio. PWV: pulse wave velocity. SBP: systolic blood pressure. DBP: diastolic blood pressure. MAP: mean artery pressure. PP: pulse pressure

**Supplementary Table 3:** Results of the bootstrapping analysis evaluating the direct, indirect, and total effects in a mediation model. Β coefficients and related 95% bias-corrected confidence intervals (C.I.) were calculated using the product of coefficients strategy and tested using the bootstrapping method (N=5000).

| Independent | Moderator | Dependent | Direct effect (β, 95% C.I.) | Indirect effect (β, 95% C.I.) | P |
| --- | --- | --- | --- | --- | --- |
| Neck circumference | Brachial MAP | Carotid stiffness | 0.037 (0.009 – 0.065) | 0.029 (0.017 – 0.043) | 0.009 |
| Neck circumference | Brachial MAP | Carotid-femoral PWV | 0.037 (0.001 – 0.072) | 0.024 (0.012 – 0.039) | 0.042 |
| Neck circumference | Carotid PP | Carotid stiffness | 0.034 (0.007 – 0.062) | 0.032 (0.019 – 0.046) | 0.015 |

PWV: pulse wave velocity. MAP: mean artery pressure. PP: pulse pressure
